# High-Masking Autism or Schizotypal Personality Disorder? Challenges in Differential Diagnosis

**DOI:** 10.1192/j.eurpsy.2026.10982

**Published:** 2026-07-31

**Authors:** K. Akhobadze

**Affiliations:** David Tvildiani Medical University, Tbilisi, Georgia

## Abstract

**Introduction:**

Autism Spectrum Disorder (ASD) is often misdiagnosed as a personality disorder in adulthood, particularly Cluster A conditions such as Schizotypal Personality Disorder (StPD). This is especially common in high-masking adults, often women, whose traits can resemble schizotypal eccentricity.

**Objectives:**

This review aims to clarify the shared and distinguishing features of high-masking ASD and StPD, explore factors that might cause misdiagnosis, and suggest ways to improve accurate identification.

**Methods:**

A literature review was conducted using PubMed, focusing on adults aged 18–55 years, both genders, and studies investigating clinical, social interaction, cognitive, and masking characteristics in ASD and StPD.

**Results:**

The literature reveals significant overlap between ASD and StPD, particularly in negative and interpersonal traits like social withdrawal, limited friendships, and idiosyncratic communication (Poletti & Raballo Schizophr Res 2020; 223,53–58). In high-masking ASD, scripted or rigid social behavior may seem eccentric, and intense, niche interests can mimic schizotypal traits (Parvaiz et al. BMC Psychiatry 2023; 23(1)). That is why diagnostic delay is so common: in a study of 161 adults with ASD, the correct diagnosis was often delayed on average by 11 years. Notably, 33.5% had never received any diagnosis, while the rest had been misdiagnosed, with personality disorders among the most frequent (Fusar-Poli et al. Eur Arch Psychiatry Clin Neurosci 2020; 272(2),187–198).

Despite similarities, there are key differences. ASD arises in early childhood with a stable course, has repetitive behaviors and sensory issues. Schizotypal traits emerge later, may progress to psychosis, and are linked to paranoia and self-identity disturbance (Poletti & Raballo Schizophr Res 2020; 223,53–58). In fact, neuroimaging (fMRI) also differentiates them: ASD shows hypoactivation of social brain regions, while StPD shows hyperactivation in the amygdala and related networks, consistent with the “hypo- vs. hyper-mentalizing” model (Stanfield et al. Schizophr Bull 2017; 43(6),1220–1228).

Gender complicates the diagnostic process even more: women with ASD often mask symptoms and are overlooked because of male-normed tools such as ADOS-2, contributing to frequent misdiagnosis as personality disorders. Misdiagnosis is also caused by gaps in developmental history and diagnostic overshadowing (Fusar-Poli et al. Eur Arch Psychiatry Clin Neurosci 2020; 272(2),187–198).

**Conclusions:**

To improve accuracy in diagnosis of ASD and StPD, clinicians need a structured approach that considers developmental history, identifies masking strategies, recognizes diagnostic bias, and distinguishes cognitive from perceptual features (Fusar-Poli et al. Eur Arch Psychiatry Clin Neurosci 2020; 272(2),187–198). Using standardized assessments and providing tailored interventions are key to ensuring accurate diagnosis and appropriate care.

**Disclosure of Interest:**

None Declared

